# PVDF Membrane Morphology—Influence of Polymer Molecular Weight and Preparation Temperature

**DOI:** 10.3390/polym9120718

**Published:** 2017-12-15

**Authors:** Monika Haponska, Anna Trojanowska, Adrianna Nogalska, Renata Jastrzab, Tania Gumi, Bartosz Tylkowski

**Affiliations:** 1 Departament d’ Enginyeria Química, Universitat Rovira i Virgili, Av. dels Països Catalans 26, 43007 Tarragona, Spain; monika.haponska@urv.cat (M.H.); adrianna.nogalska@urv.cat (A.N.); tania.gumi@urv.cat (T.G.); 2 Faculty of Chemistry, A. Mickiewicz University, Umultowska 89b, 61-614 Poznan, Poland; renatad@amu.edu.pl; 3 Centre Tecnològic de la Química de Catalunya, Carrer de Marcel·lí Domingo, 43007 Tarragona, Spain

**Keywords:** PVDF membrane, coagulation bath temperature, polymer molecular weight

## Abstract

In this study, we successfully prepared nine non-woven, supported polyvinylidene fluoride (PVDF) membranes, using a phase inversion precipitation method, starting from a 15 wt % PVDF solution in *N*-methyl-2-pyrrolidone. Various membrane morphologies were obtained by using (1) PVDF polymers, with diverse molecular weights ranging from 300 to 700 kDa, and (2) different temperature coagulation baths (20, 40, and 60 ± 2 °C) used for the film precipitation. An environmental scanning electron microscope (ESEM) was used for surface and cross-section morphology characterization. An atomic force microscope (AFM) was employed to investigate surface roughness, while a contact angle (CA) instrument was used for membrane hydrophobicity studies. Fourier transform infrared spectroscopy (FTIR) results show that the fabricated membranes are formed by a mixture of TGTG’ chains, in α phase crystalline domains, and all-TTTT trans planar zigzag chains characteristic to β phase. Moreover, generated results indicate that the phases’ content and membrane morphologies depend on the polymer molecular weight and conditions used for the membranes’ preparation. The diversity of fabricated membranes could be applied by the End User Industries for different applications.

## 1. Introduction

Polyvinylidene fluoride (PVDF) is one of the most widely studied and well-accepted polymers for membrane fabrication through a conventional phase inversion method (PIP). PVDF-unique features include good degradation resistance against radiation, outstanding chemical and thermal resistance, and excellent mechanical properties [1,2,3]. It is a specialty plastic used in a vast number of traditional, well-defined applications, such as piping and tubing, membranes, cables, and as an insulator for premium wire. There are also emerging applications, such as lithium-ion batteries, coatings for new energy and electronic devices, photovoltaic films, and medical applications [4,5,6,7]. To meet booming global demand for this thermoplastic polymer in energy-efficient, environmental and industrial applications, very recently (November 2017) Solvay S.A. Company—one of the industrial leaders in PVDF production—inaugurated its Solef^®^ polyvinylidene fluoride (PVDF) plant in China [8]. According to a recently published report [9], mainly ten manufacturers dominate the PVDF global market, and research and development has been among the key points in the design of PVDF final products. Depending on the polymerization process (emulsion polymerization, suspension polymerization, etc.), PVDFs with different molecular weight distributions are available, which is a critical factor for the final performance of prepared membranes [10]. It is well-known that the molecular weight of the polymer has a significant effect on the rheology of the polymer solution, as well as on the thermodynamic and kinetic aspects of the phase inversion, which influences the structure and performance of the final membranes [11]. Knowledge of polymer crystallinities and their resulting membrane morphologies is important as a basis of understanding the polymer’s membrane permeability and selectivity, as well as its various chemical and mechanical properties [12]. Researchers have investigated the influence of an addition of a small concentration of various components on membrane structure. These additives often show specific interactions with one of the other three components. Indeed, a number of publications focused on composite PVDF membranes have been published during the last decade [4,13,14,15,16]. However, different methods and strategies have been applied for the PVDF polymers production, which could impact their polymorphs [17,18,19]. Thus, to design new membranes and gain innovative ideas, a fundamental knowledge about the membrane obtained from current commercially available PVDF polymers is highly required. For this reason, we have investigated the influence of non-solvent bath temperature on PVDF membrane polymorphism and morphologies. In our study, the coagulation bath temperatures were carefully selected and varied in a range of 20–60 °C. The membranes were prepared by applying the PIP method, in which a room temperature casting solution (20 °C) was used for membrane preparation, unlike the previously reported protocols [20,21]. The lowest selected temperature (20 °C) of the coagulation baths corresponded to room temperature, in which most of the reported membranes have been fabricated [20,22], while 60 °C was the highest temperature in which we were able to control the following parameters: (a) the non-solvent temperature homogeneity in the whole volume of the coagulation bath, and (b) the non-solvent evaporation. A 40 °C temperature was selected as a medium one. In addition, the influence of different PVDF molecular weights on membrane morphologies and polymorphism was investigated.

## 2. Materials and Methods 

### 2.1. Materials

PVDF powders Solef 6010, Solef 1015, and Solef 6020 were kindly donated by Solvay Specialty Polymers (Bollate, Italy). *N*-methyl-2-pyrrolidone (NMP, 99%) was provided by Panreac (Inca, Spain). Non-woven Hollytex 34 GR was provided by STEM Company (Teijin Aramid, Arnhem, The Netherlands). Distilled water was used as a non-solvent in the coagulation bath.

### 2.2. Preparation of PVDF Membranes

PVDF membranes were prepared by the immersion precipitation method. In brief, PVDF pellets were dissolved in NMP at 80 °C, with vigorous stirring for 48 h, to form a 15 wt % homogeneous casting solution. After air bubbles were removed completely, the resulting solution was cooled to room temperature, 20 ± 2 °C, and spread uniformly onto a glass plate with a non-woven support (15 cm × 20 cm) attached, using a casting knife with a 250-μm gate opening (K Paint Applicator, R K Print Coat Instruments, Ltd., Litlington, UK) and the coating speed set up at 2 m/min. Then the membranes were immersed immediately (approximately 10 s after coating) into a precipitation bath of deionized water (DW, 3 L) set up at the following temperatures: 20 ± 2 °C, 40 ± 2 °C, or 60 ± 2 °C. The membranes were kept in the non-solvent bath for 20 min to complete the film solidification. To maintain the same precipitation conditions, and to avoid the non-solvent contamination by solvent, fresh DW was used for each membrane fabrication process. The formed solid membranes were washed thoroughly with deionized water to remove residual NMP and dried at about 40 °C for 24 h under vacuum before further characterization.

### 2.3. Membrane Characterization

The crystalline forms of the PVDF membranes were investigated by Fourier transform infrared spectroscopy (FTIR), using VERTEX 70 (Bruker, Poznan, Poland) equipped with a Platinum-ATR-accessory with 2 cm^−1^ resolution and 32 scans. Obtained FTIR-ATR results were assigned to the top surface of the PVDF membranes. According to literature [23], the depth of penetration for the PVDF membrane, using the 45° of the incident angle of light on an ATR element (Ge crystal) similar to the equipment used here, is approximately 0.44–0.95 µm. The wavelength range is 700 to 1500 cm^−1^. As is described above, the M1–M9 composite membranes were fabricated using the PIP method, by casting the PVDF polymeric solutions on the non-woven polyester support. Due to the membrane composition (the bottom part formed by the non-woven support, and the top part formed by the PVDF film), the FTIR-ATR studies were carried out only on the top membrane surfaces (PVDF side). It is well known that IR spectroscopy in conventional transmission mode is a useful tool to define the composition, structure and conformation of the polymeric chains, but it cannot provide sufficient information on the structures of polymeric porous membranes. The porous membranes are almost opaque in the fingerprint region (1500–400 cm^−1^), due to their porosity and thickness. This porous structure possesses a very large and irregular air/solid interface, which greatly scatters the photons (especially for the higher wavenumbers) causing a remarkable slope and low signal-to-noise ratio. Thus, for the investigated composite porous PVDF membranes, the IR measurements in transmission mode are less useful for yielding crystalline information out of the overall membranes. This means that to study the fingerprint region of PVDF-based porous membranes, the surface sensitive FTIR-ATR spectroscopic technique must be used.

The cross-sections and surface morphologies of the fabricated PVDF membranes were characterized by Environmental Scanning Electron Microscopy (Quanta 600, FEI, Thermo Fisher Scientific, Barcelona, Spain) [24]. In order to prepare the samples for cross-section studies, first the membranes were wet with ethanol. Then they were immersed in liquid nitrogen, and broken at −270 °C. The membrane pore size was calculated using the environmental scanning electron microscope (ESEM) micrographs and IFME software (Thermo Fisher Scientific, Barcelona, Spain) [25].

An Agilent 5500 Environmental Atomic Force Microscope (AFM, Agilent Technology, Barcelona, Spain) equipped with an extender electronics module was used to investigate membrane surface morphologies. The AFM micrographs (2 × 2 μm^2^) were captured at room temperature in tapping mode, using Multi 75 (BugetSensors, Sofia, Bulgaria) silicon cantilevers (length = 225 μm, width = 28 μm, and thickness = 3 μm), with a force constant of 3 N/m, 75 kHz resonance frequency, and 0.7–2 Hz scan rate. During the AFM experiments, the studied samples were located on an active vibration isolation chamber (Agilent Technology, Barcelona, Spain), which protects them from external vibration and eliminates external noise. The generated images were analyzed by The Nanotec WSxM 5.0 Develop 4.0 software (NanotecElectronica S.L, Madrid, Spain).

Contact angles (CA) of 3 µm drops of milli-Q water on membrane surfaces were investigated by using a Dataphysics OCA 15EC (DataPhysics Instruments GmbH, Filderstadt, Germany) apparatus at room temperature. The contact angle measures were carried out immediately after putting the water drop on the membrane surface. The experiment was repeated five times, using different areas of the membrane, and average CA values are provided. 

**Scheme 1:**
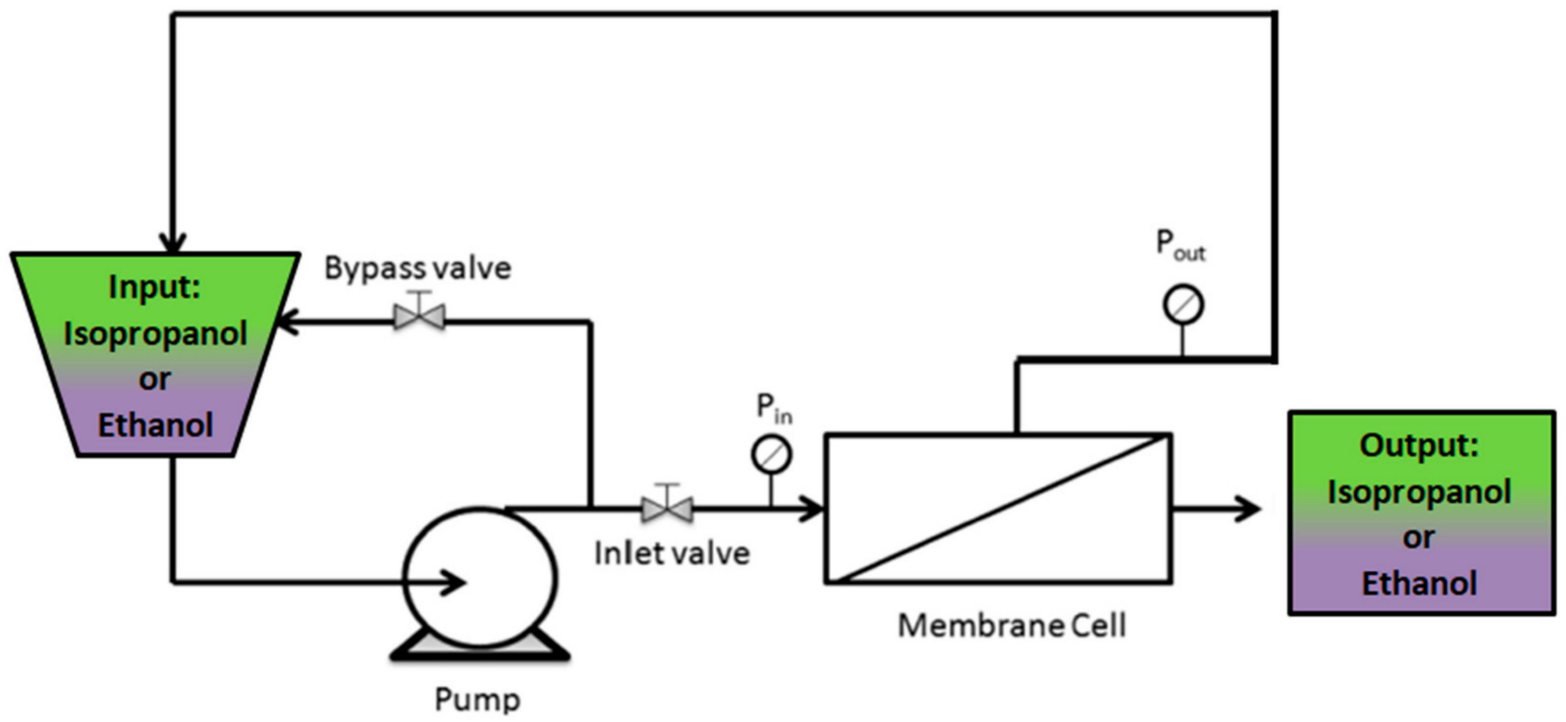
Flow diagram of the cross-flow nanofiltration apparatus.

Permeability experiments through the investigated membranes were performed with ethanol and isopropanol as organic solvents, employing a custom-made, stainless steel, cross-flow nanofiltration device equipped with a disk membrane module, as shown in Scheme 1. The experiments were carried out at 5 bar and at room temperature. The effective membrane area in the module was 12.6 cm^2^. In order to compact the membrane, the system was run with the organic solvents at 5 bar for 30 min prior to permeability measurements. The flux (*J*) through the membrane was calculated by the following equation:

$$J=\frac{V}{A\cdot \Delta t}$$

where *V* is the output organic solvent volume (L), *A* is the membrane area (m^2^), and Δ*t* (h) is the permeation time.

## 3. Results and Discussion

It is well-known that PVDF crystals have three different molecular conformations: trans-gauche-trans-gauche’ (TGTG’), trans-trans-trans (TTT), and trans-trans-trans-gauche (TTTG). They also have five different polymorphs: α (phase II), β (phase I), γ (phase III), δ, and ε [15,26]. Each of the polymorphs possesses its own unique properties, which could affect the morphology and performance of the fabricated membrane. It has been found that β-phase PVDF has some specific properties, such as polarity and higher mechanical strength, compared with α-phase [4]. As is shown in Table 1, we prepared nine membranes, named M1–M9, applying the phase inversion precipitation method, with NMP as a solvent and distilled water as a non-solvent, at different temperatures of coagulation bath. Membranes M1, M4, M7 were precipitated at 20 ± 2 °C, while membranes M2, M5, M8, and membranes M3, M6, M9 were obtained at 40 ± 2 °C and 60 ± 2 °C, respectively. Furthermore, membranes M1–M3 were done using PVDF with 300–320 kDa molecular weight, while membranes M4–M6 and M7–M9 were fabricated using PVDF with 570–600 kDa and 670–700 kDa, respectively. To verify the influences of (1) coagulation bath temperature and (2) molecular weight of PVDF on the polymorphism of the investigated membranes, infrared spectroscopy, which is a common method for PVDF crystalline phase characterization [26], was carried out.

**Figure 1:**
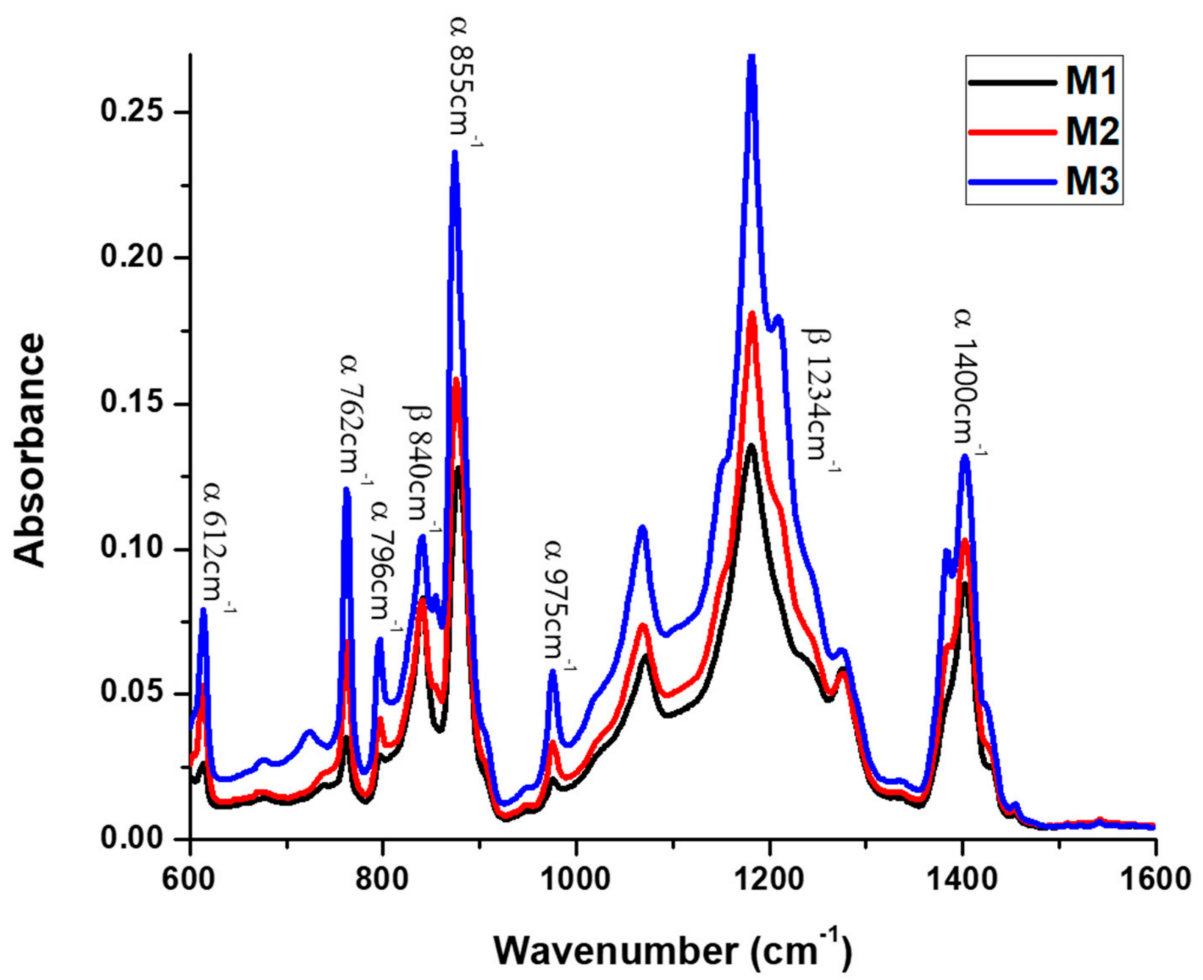
FTIR-ATR spectra of characteristic peaks for α and β phases of the top membrane surfaces of M1, M2, and M3, prepared from the same molecular weight of polyvinylidene fluoride (PVDF) at different temperature.

**Figure 2:**
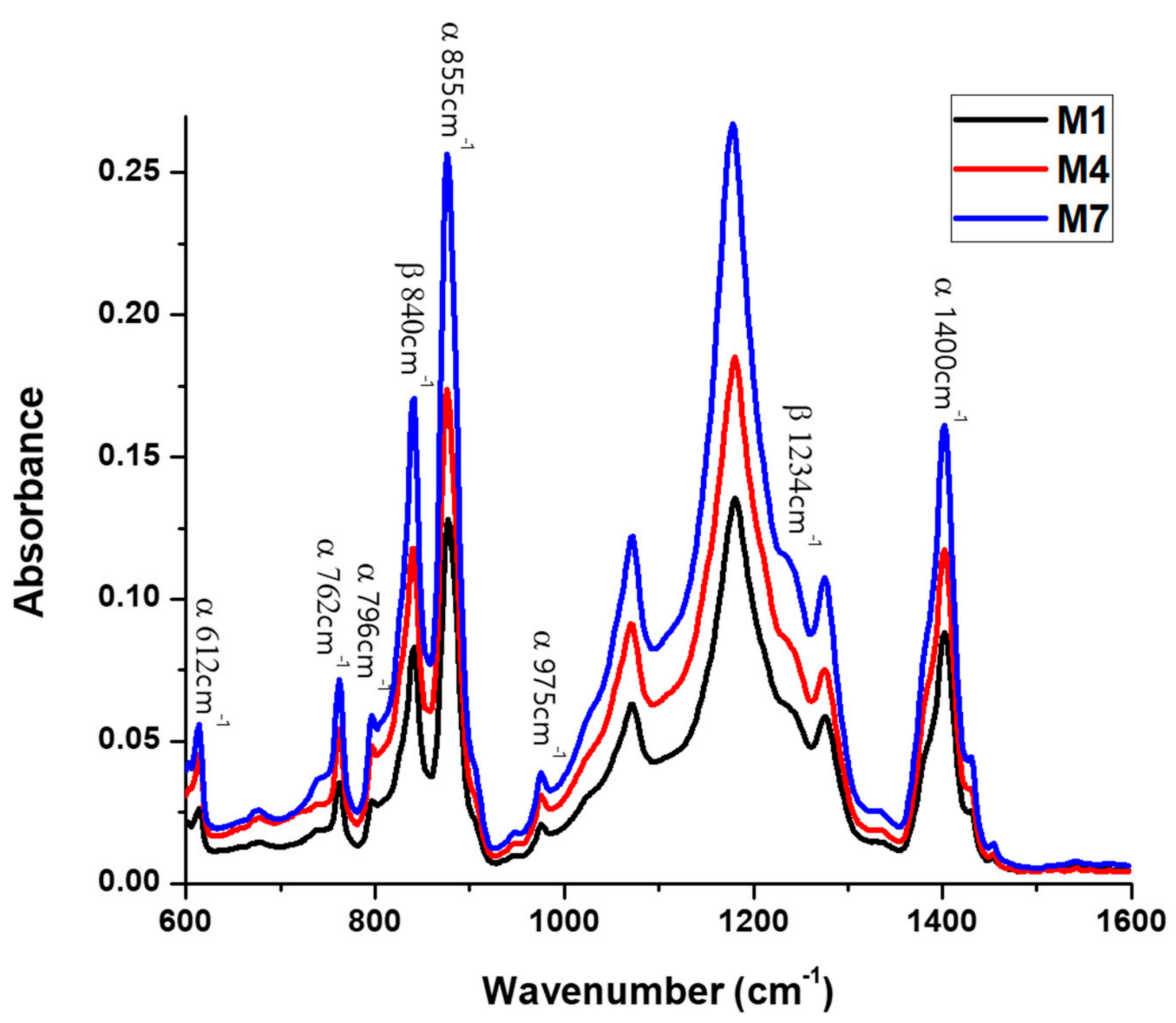
FTIR-ATR spectra of characteristic peaks for α and β phases of the top membrane surfaces of M1, M4, and M7, prepared at the same temperature using PVDF with different molecular weights.

**Figure 3:**
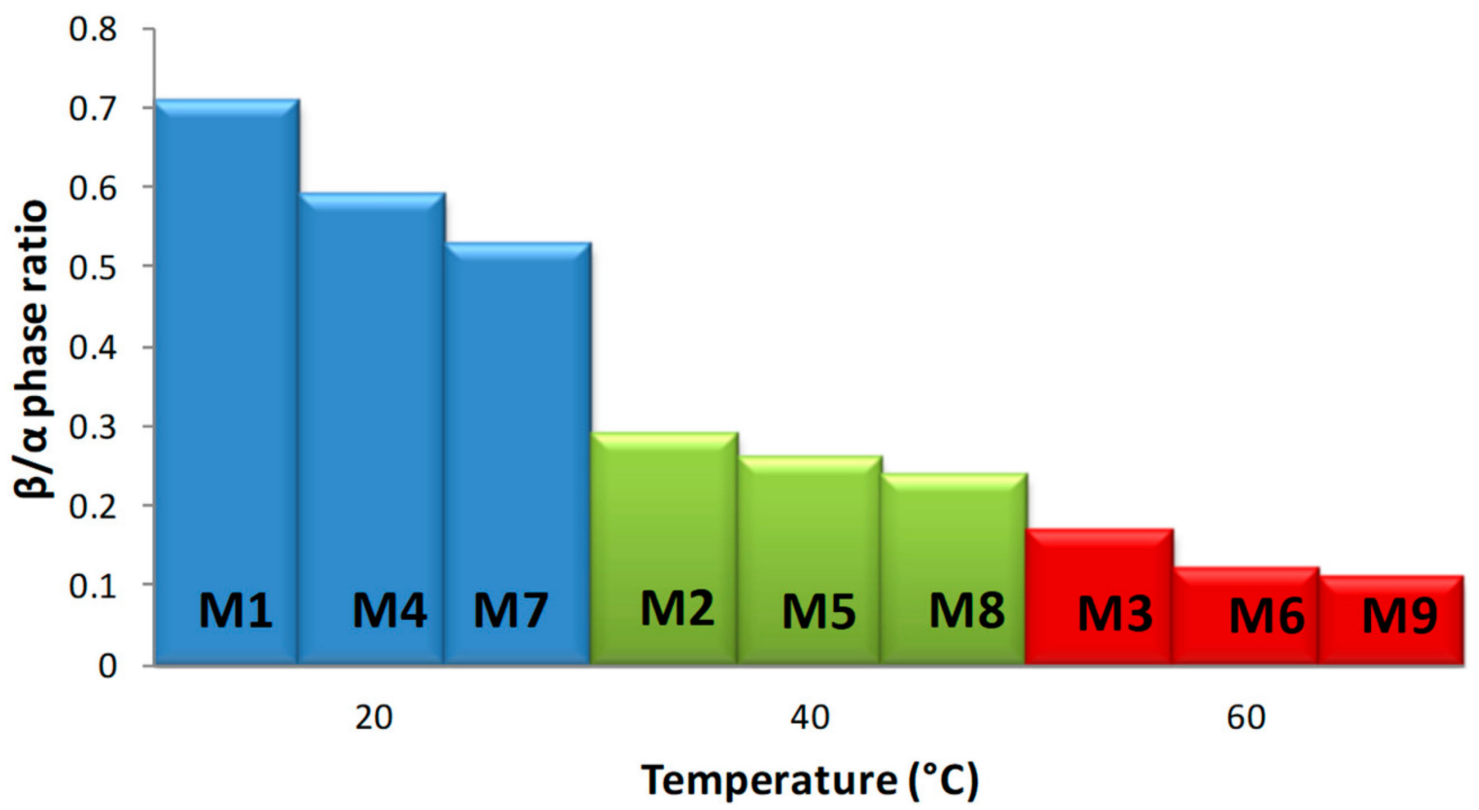
β/α phase ratios of M1–M9 membranes.

Figure 1 shows the FTIR-ATR spectra of M1, M2 and M3 top membrane surfaces, fabricated from 15 wt % PVDF 300–320 kDa polymeric solution, at 20, 40, and 60 °C, respectively, while Figure 2 shows the spectra of M1, M4, and M7 membranes, precipitated at 20 °C from 15 wt % PVDF solutions with molecular weights of 300–320 kDa, 570–600 kDa, and 670–700 kDa, respectively. As has been reported in literature [22,27], the presence of representative bands at 612, 762, 796, 855, 975, and 1400 cm^−1^ indicates the formation of α phase, while the vibration bands at 840 and 1234 cm^−1^ suggest the existence of the orthorhombic β phase. Furthermore, the absence of bands at 833 and 1233 cm^−1^ excludes the γ phase. Furthermore, and due to the consistency of the C–F stretch at 1182 cm^−1^, the PVDF membranes have good stability during the thermal and chemical treatments [16]. The FTIR-ATR results suggest that the investigated membranes are formed by a mixture of TGTG’ chains, in α phase crystalline domains, and all-TTTT trans-planar zigzag chains characteristic to β phase. To estimate α or β phase ascendency in the top membrane surface structure, β/α phase ratios were calculated for all M1–M9 membranes, and they are given in Table 1 and Figure 3. These values were calculated by comparing the absorbencies of vibration band peaks at 840 cm^−1^ (CH_2_ rocking) and 762 cm^−1^ (CF_2_ bending and skeletal bending) using the following equation [23]:

(1) $$\frac{F\left(\beta \right)}{F\left(\alpha \right)}=\frac{A_{\beta }^{840}}{(K_{\beta }^{840}d^{840}/K_{\alpha }^{762}d^{762})A_{\alpha }^{762}}=\frac{A_{\beta }^{840}}{1.15A_{\alpha \;}^{762}}$$

where F(β) and F(α) are mass fractions of β and α phase, and $A_{\beta }^{840}$ and $A_{\alpha }^{762}$ are the baseline-corrected absorption peaks of the β and α phases, at 840 and 762 cm^−1^, respectively. *K* and *d* represent the absorption coefficient and penetration depth at the corresponding wavenumber, respectively. The values $K_{\beta }^{840}$ = 7.7 × 10^4^ cm^2^/mol, $K_{\alpha }^{762}=6.1\times \;$10^4^ cm^2^/mol, and $d^{840}=0.79\;µm;\;d^{762}=0.87\;µm\;$ were used based on literature findings [23].

Generated results clearly demonstrate that membrane polymorphism strongly depends on the temperature of the non-solvent used for its precipitation (Figure 3). The β phase mostly dominates in the top membrane surface fabricated at 20 °C, while α phase content increases as the coagulation bath temperature increases (up to 60 °C). According to Wang et al. [28], during the immersion of the casting solutions to the non-solvent at a lower temperature (15 °C), a delayed liquid–liquid de-mixing mechanism occurs. This mechanism could encourage a gelation process, indicated by the formation of micro-crystallites and the β phase. On the other hand, the temperature increase favors the mobility of PVDF chains, which facilitates the formation of the stable non-polar α phase with a monolithic lattice structure. These observations are consistent with literature findings; Gradys and Sajkiewicz [29] stated that the thermodynamically less stable β phase is typically formed at room temperature, while Gregorio [30] reported that the α phase normally forms at high temperature. Moreover, comparing the values of the β/α phase ration given in Table 1, for the membranes formed at the same temperature but from different molecular weight PVDF solutions (i.e., M1–M4–M7, M2–M5–M8, and M3–M6–M9), the increase of β phases can be observed with molecular weight decrease. We think that this could take place due to significant polymer chain entanglements at lower molecular weights, which lead to the oriented packing of CH_2_–CF_2_ dipoles, thus supporting the formation of β phase crystals.

**Figure 4:**
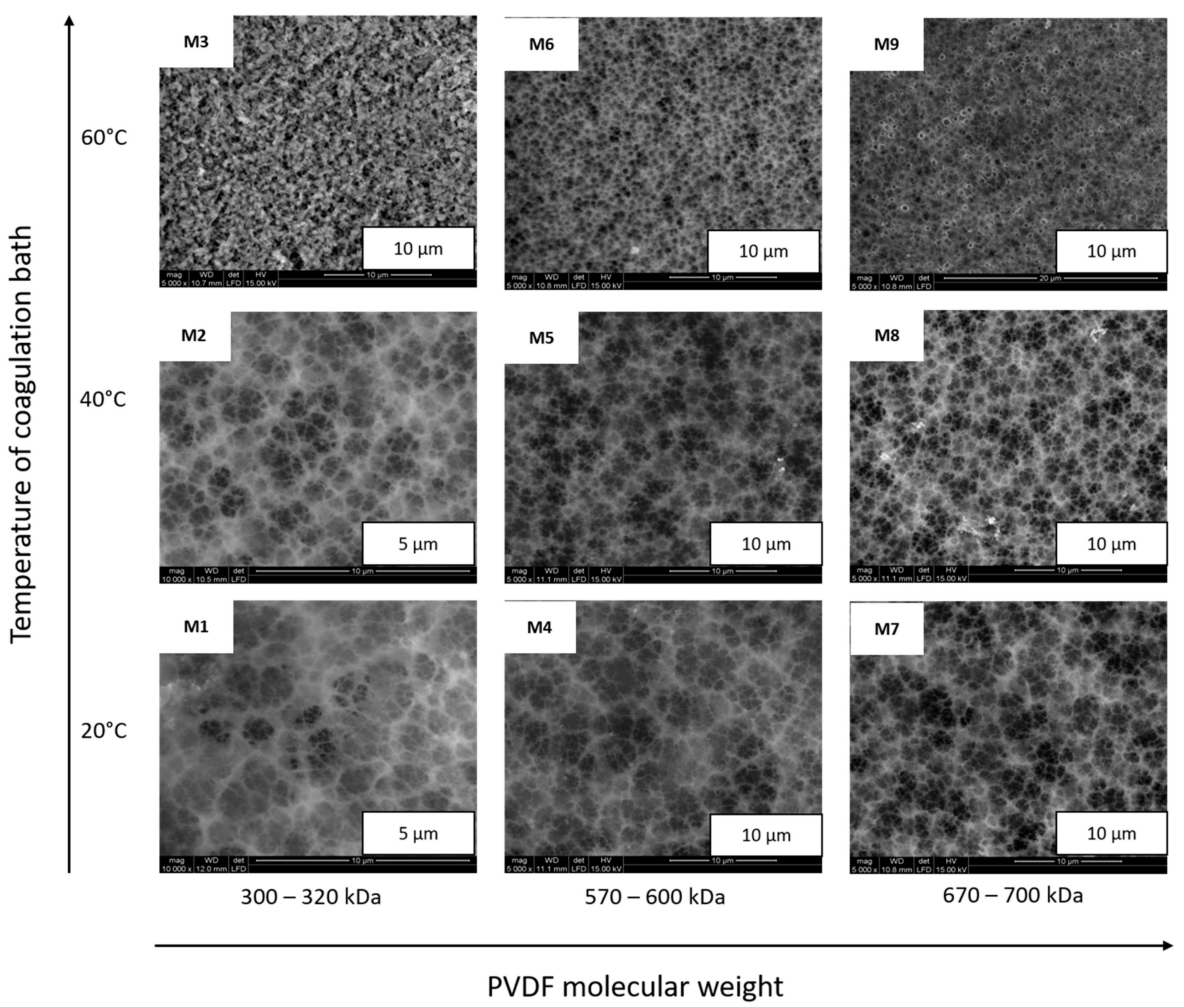
Environmental scanning electron microscope (ESEM) micrographs of the M1–M9 membrane top surfaces.

**Figure 5:**
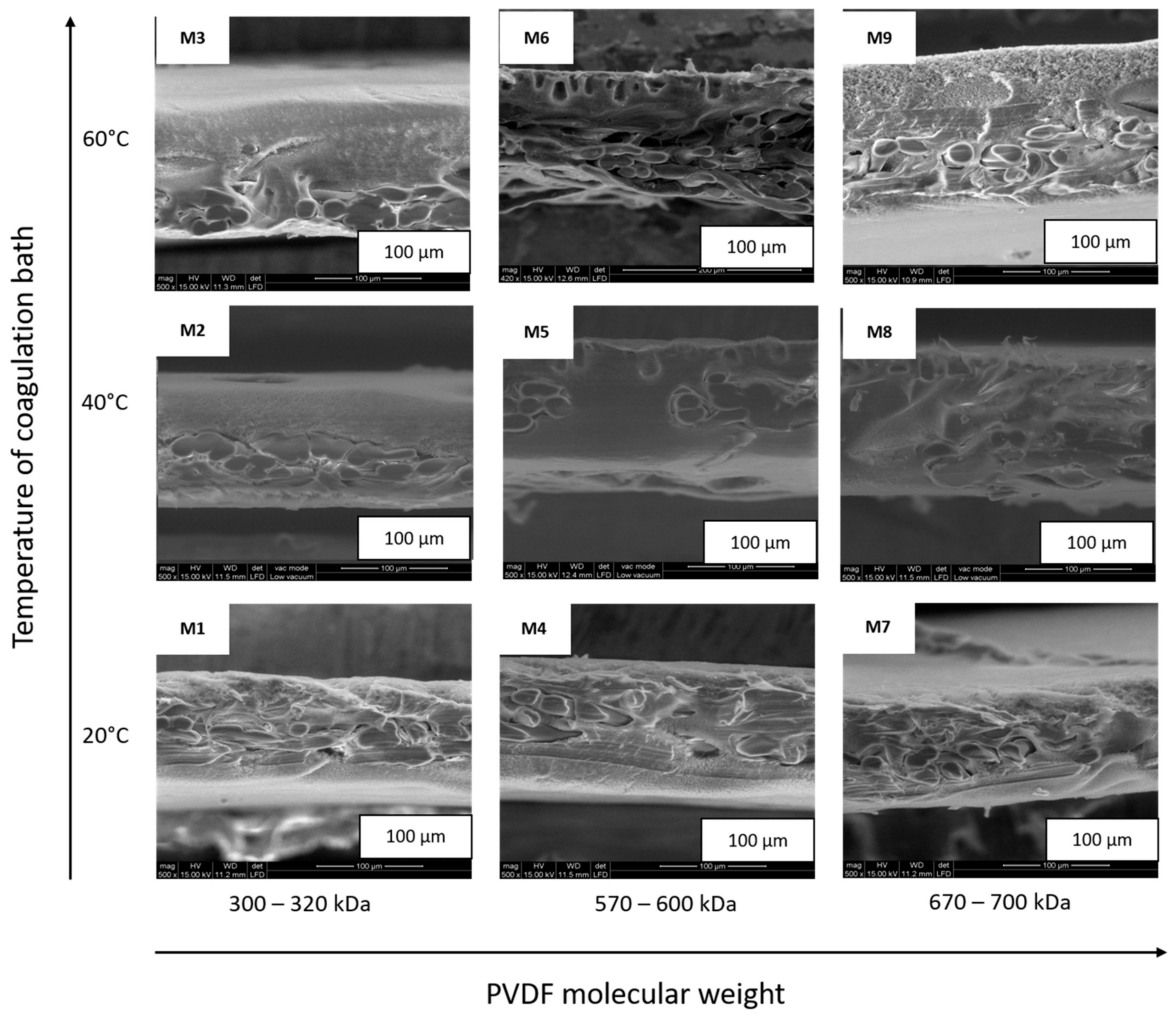
ESEM micrographs of the M1–M9 membrane cross-sections.

Figure 4 shows the ESEM micrographs of the M1–M9 membrane top surfaces, while Figure 5 provides their cross-sections. Membrane thicknesses, calculated by Image-ProPlus 5 software on ESEM cross-section micrographs [31], are in a range of 103–120 ± 2 μm (see Table 1). According to literature [32], membrane thickness usually decreases with the increase of polymer viscosity (modulated by polymer molecular weight); however, in our studies, this trend has not been registered, probably due to the presence of the non-woven support, whose structure is well filled by the polymers and forms an integrated part of the membranes. As can be observed in Figure 4, membranes M1, M4, and M7, prepared at 20 °C, possess three-dimensional fibriform network morphologies. In our opinion, this is due to the PVDF semi-crystallinty. Indeed, Wang et al. [28] observed that at low temperatures, the β phase micro-crystallites connect various polymeric chains together, and form these 3D fibriform networks, similar to those demonstrated in Figure 4. Moreover, the ESEM investigation shows that the PVDF membrane surface morphology is strongly influenced by the temperature of the coagulation bath. Thus, surface porosity was verified by providing pore mean size distributions, which were calculated using IFME software, based on the ESEM surface micrographs [25]. As is presented in Table 1, the mean pore size measured for the M1–M9 membrane top surfaces is in a range of 0.18–0.49 ± 0.26 µm, and its value is related to the temperature of the non-solvent in which the membrane was precipitated, as well as to the molecular weight of the polymer used for membrane fabrication. Comparing these values to those given in Table 1, we can conclude that with an increase in temperature and molecular weight, the mean pore size decreases, which is in accordance with literature data. Cardoso et al. [33] stated that increasing water bath temperature leads to less porous membrane surfaces. The effect of PVDF molecular weight on surface porosity was studied by Hassankiadeh and co-authors [34], who have investigated PVDF hollow fiber membranes using PVDF Solef 1015 and 6020, with molecular weight ranges of 570–600 kDa and 670–700 kDa, respectively. The authors reported that the mean pore size of the fiber surfaces decreases as the PVDF molecular weights increase. Matsuyama and co-authors [35] reported that reducing the viscosity of the polymer solution by decreasing the polymer molecular weight helps the solvent displacement. This provides the possibility of improved symmetry of the membrane structure in the lower-molecular-weight polymer, compared with that in the higher-molecular-weight polymer, which has a significant impact on membrane porosity and mean pore size. Further inspection of the ESEM images reveals that the membrane cross-section morphologies (Figure 5) are strongly affected by the polymer’s molecular weight. As can be observed in the micrographs, membranes M2 and M3 possess a compacted structure, while membranes M4 and M5 possess drop-like macro-voids at the top part, and dense sponge-like structures at the bottom part. Membranes M7 and M8 possess mostly spongy-like structures. From the kinetic point of view, the increase in viscosity with an increase in the PVDF molecular weight decreases the solvent/non-solvent exchange rate, by increasing kinetic hindrance in the phase inversion process. Thus, delayed de-mixing is endorsed, which results in the formation of a less drop-like structure and a more sponge-like structure. The drop-like structures are predicted to be formed in the intermediate range of molecular weight, as a result of the superimposition of the thermodynamic and the kinetic effect, if the thermodynamic effect controls the phase inversion process earlier than the kinetic effect does [36].

**Figure 6:**
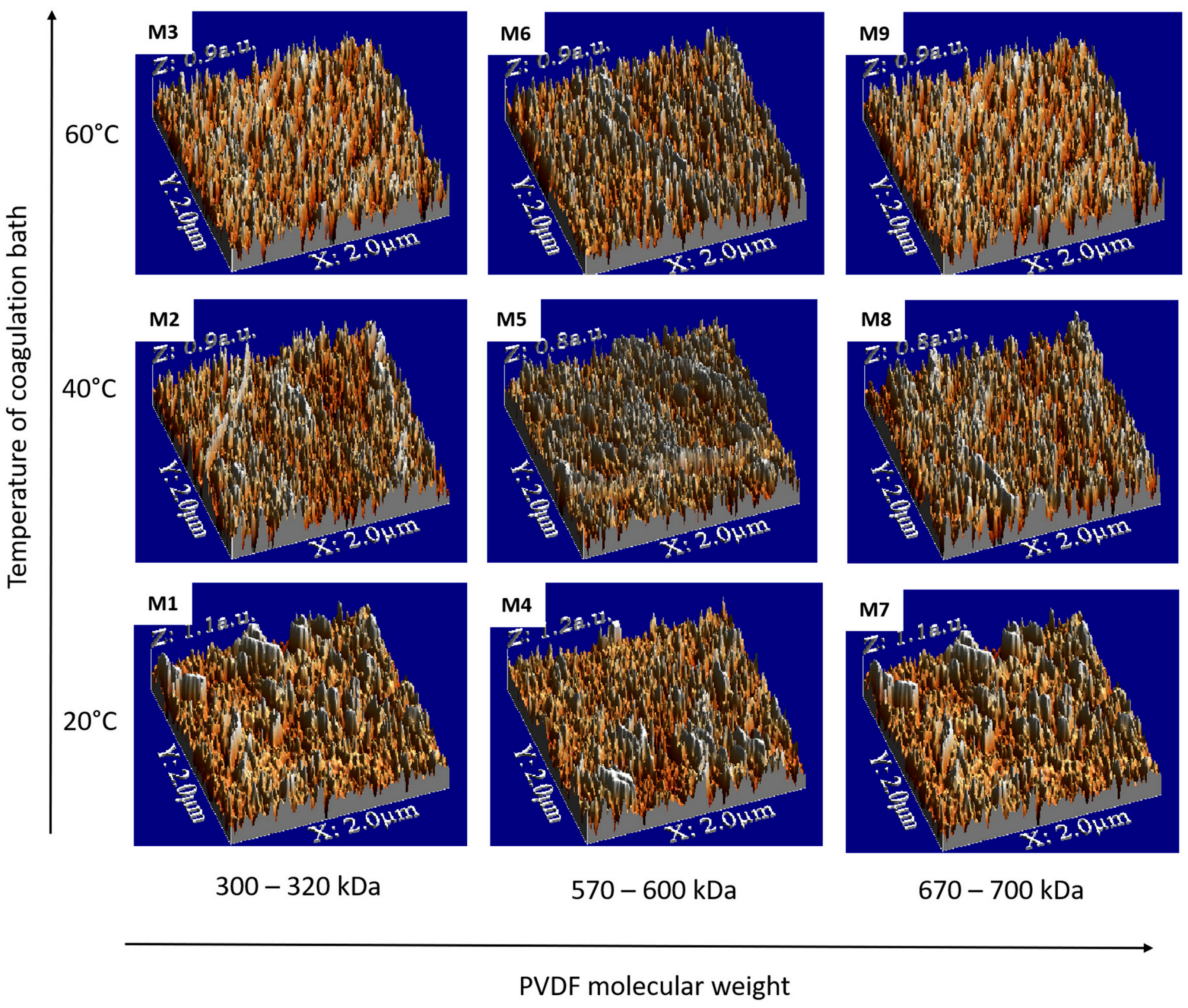
Atomic force microscope (AFM) images of M1–M9 membrane top surfaces.

The surface roughness of the membranes has important consequences on transport phenomena, and has been the topic of numerous investigations [2,34,37]. Figure 6 shows the AFM images of the top surface of the M1–M9 PVDF membranes, while the root mean square (RMS) roughness, considered as a standard deviation of height, is listed in Table 1. Comparing the RMS values, it can be seen that the smoother membranes were obtained at 40 °C. This behavior could occur due to the solvent/non-solvent de-mixing at the appropriate kinetic rate, leading to completion through incorporation of PVDF chains into the non-woven support matrix. These results are of current interest for technological applications, where smooth, uniform, homogeneous, and porous membranes are needed, such as scaffolds for cell growth or as a separator of lithium ion batteries [38]. It is commonly known that the roughness parameter is linked to the contact angle of the membrane and its wettability [39]. As has been reported in literature [25,39,40], surface roughening tends to increase the contact angle values. Table 1 provides the static contact angle results measured on the M1–M9 membranes. Indeed, the lowest value (59.0° ± 2.8°) was measured for the smoothest M5 membrane, prepared at 40 °C, while the highest value (98.1° ± 5.9°) was measured for membrane M9, which had the highest RMS value.

**Figure 7:**
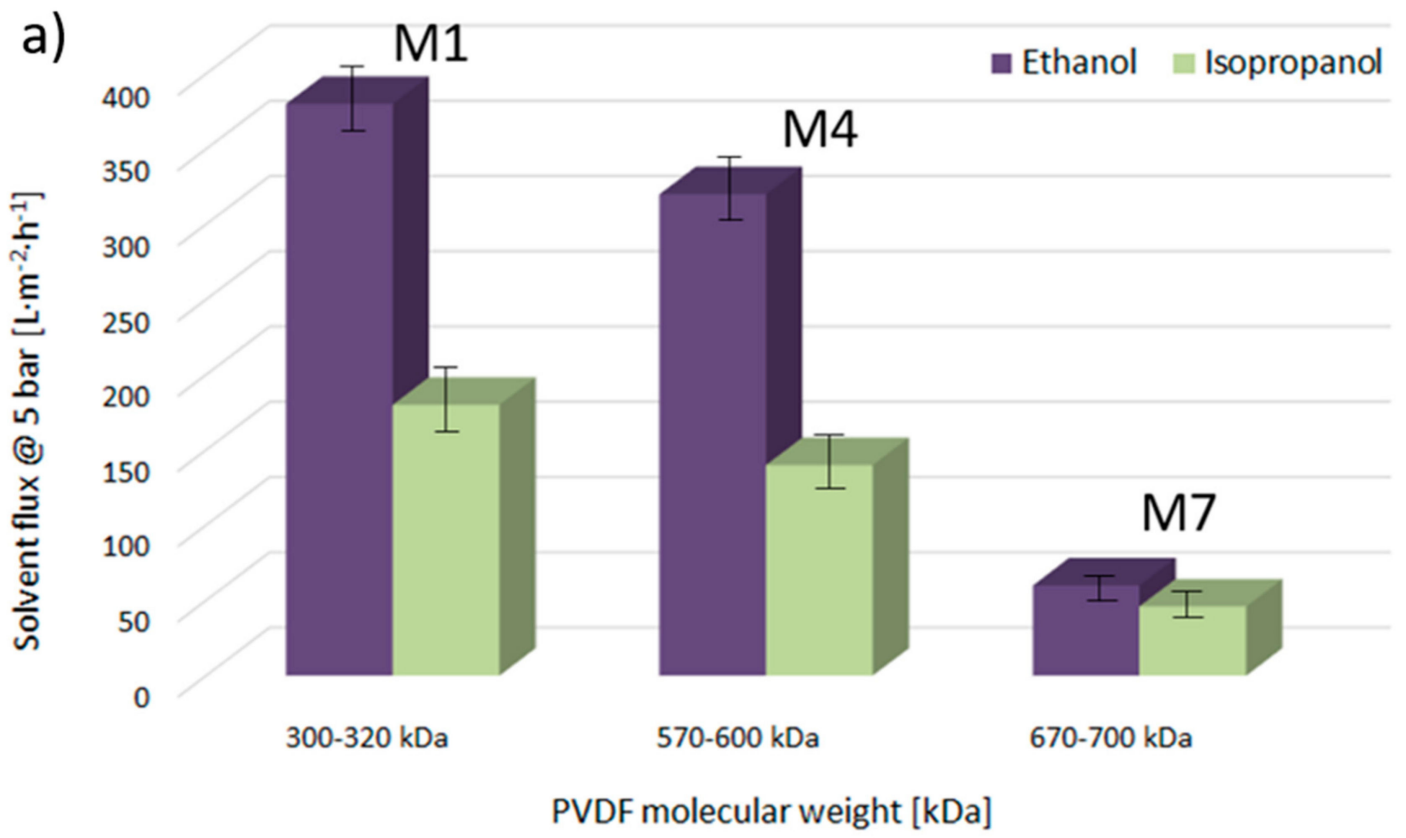

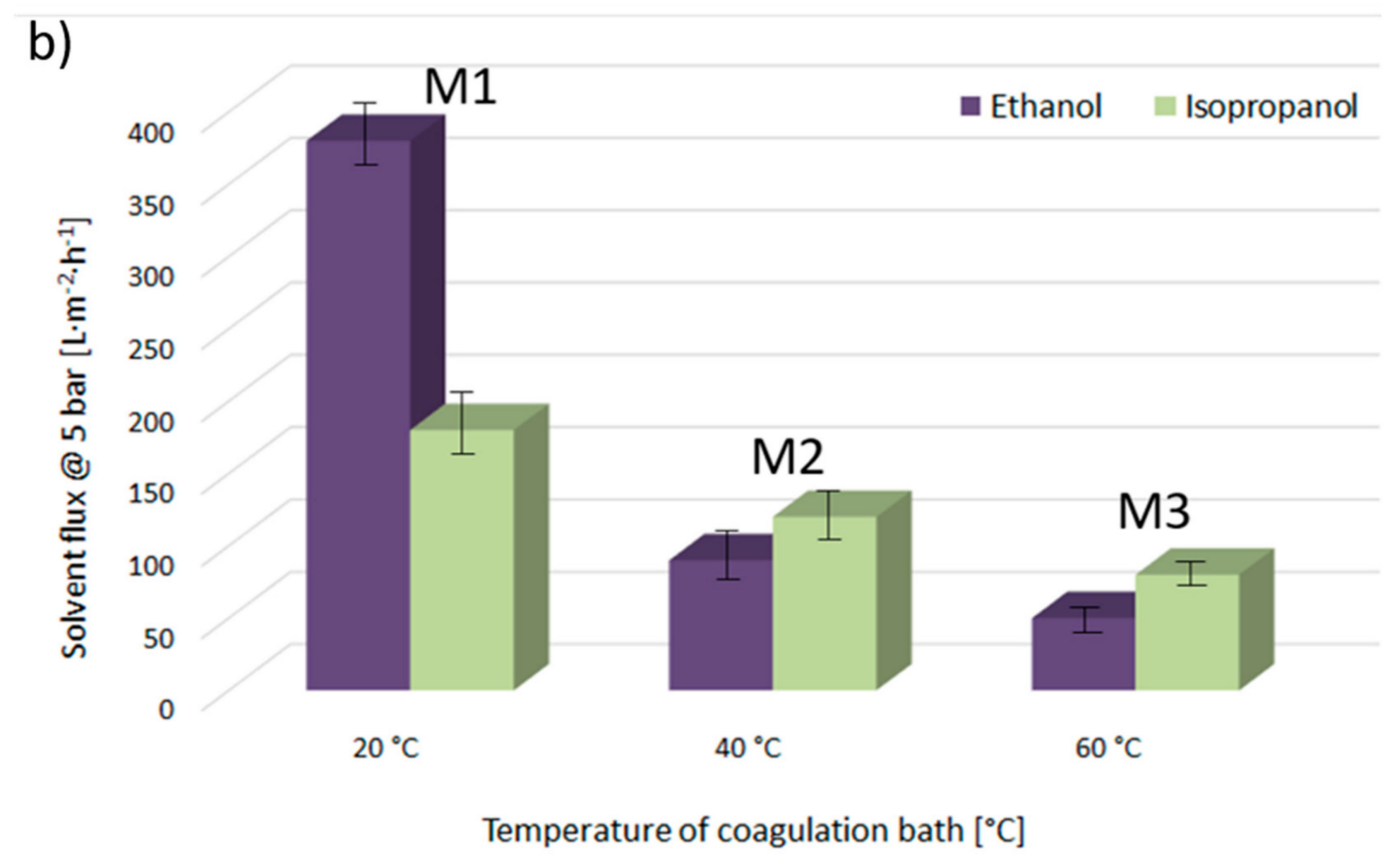
Ethanol and isopropanol fluxes through investigated membranes at 5 bar and room temperature.

Figure 7a provides ethanol and isopropanol flux values, measured at 5 bar and at room temperature through the M1, M4, and M7 membranes, precipitated at 20 °C from the PVDF solutions, with molecular weights of 300–320 kDa, 570–600 kDa, and 670–700 kDa, respectively. Figure 7b gives the flux values for the same solvents passed through the M1, M2, and M3 membranes, fabricated from the PVDF 300–320 kDa polymeric solution at 20, 40, and 60 °C, respectively. First of all, it is worth highlighting that the fluxes of organic solvents decrease with the decrease of membrane pore diameter, irrespective of the type of solvent used. Then, it is important to note that the transport mechanism of non-aqueous systems through a polymeric membrane is strongly influenced by the structures and properties of the solvents. It has been reported that the membrane–solvent interactions can be expected to vary with respect to the solvent properties, such as viscosity, dielectric constant, molecular size, dipole moment, solubility parameter, and surface tension [31]. Indeed, comparing the flux values for isopropanol and ethanol, it can be observed that in the case of the membranes M1, M4, and M7, where the β-phase with polar zigzag (all-trans) conformation dominates in the membrane surface structures, the flux of pure ethanol (relative polarity 0.654, [41]) is higher than that of less polar isopropanol (relative polarity 0.546). Moreover, it has been reported that the flux values decrease with increasing molecular length, i.e., by lengthening the alcohol structure with additional CH_2_ groups, irrespective of the transport mechanism. Nevertheless, it is important to highlight that by comparing the flux values across the membranes M2 and M3, in which the membrane surfaces are mainly formed by the nonpolar α-phase, the flux values are slightly higher for the isopropanol than for the ethanol. These results could indicate that the transport of ethanol and isopropanol through the PVDF membranes could be partially governed by the affinity between the solvent and the membrane.

This diversity of membrane surface wettability and organic solvent permeability, as well as cross-section and surface morphologies, could find broad application in fields such as medicine, separation and concentration of biologically active compounds, etc., depending on the end user industries’ individual profiles and requirements.

## 4. Conclusions

By using phase inversion precipitation techniques, nine PVDF-based membranes were fabricated at 20, 40, and 60 °C, using three different polymer molecular weights: 300–320 kDa, 570–600 kDa, and 670–700 kDa. Based on an FTIR-ATR investigation, it was observed that membrane polymorphism is influenced by polymer molecular weight, as well as the temperature of non-solvents used for membrane precipitation. The membranes with dominated β phase are formed at lower temperatures and using PVDF with lower molecular weight. Furthermore, by ESEM investigation with IFME software, it was observed that the presence of the β phase has a significant impact on membrane morphology, and favors three-dimensional fibriform network surface structure formation. Moreover, achieved results demonstrated that cross-section membrane morphologies are impacted by PVDF molecular weight. At low molecular weights, compact membranes were produced, while at intermediate and at high molecular weights, drop-like and spongy-like membranes were fabricated, respectively. AFM and contact angle studies show that the smoother membranes were produced at 40 °C using intermediate polymer molecular weight. Permeability tests, with ethanol and isopropanol through the investigated membranes, demonstrated that the flux of the organic solvents decreases with a decrease of membrane pore diameter, irrespective of the type of solvent used. Furthermore, reported results evidenced a possible correlation between membrane polymorphism and membrane performance.

## Figures and Tables

**Table 1:** M1–M9 membrane preparation parameters, and the characteristics of the resulting membranes.

Membrane	Molecular weight of polymer [kDa]	Temperature of coagulation bath [°C]	β/α phase ratio	Main pore size [μm]	Root mean square (RMS) roughness	Contact angle [°]	Thickness of membrane [μm]
M1	300–320	20 ± 3	0.71	0.42 ± 0.01	16.07 ± 0.08	68.7 ± 3.4	106 ± 2
M2		40 ± 3	0.29	0.37 ± 0.02	12.08 ± 0.07	61.2 ± 1.0	103 ± 2
M3		60 ± 3	0.17	0.18 ± 0.03	17.18 ± 0.11	78.0 ± 5.4	106 ± 2
M4	570–600	20 ± 3	0.59	0.38 ± 0.04	15.17 ± 0.15	74.5 ± 9.0	107 ± 2
M5		40 ± 3	0.26	0.22 ± 0.03	11.26 ± 0.13	59.0 ± 2.8	120 ± 2
M6		60 ± 3	0.12	0.16 ± 0.02	16.56 ± 0.17	79.9 ± 4.9	113 ± 2
M7	670–700	20 ± 3	0.53	0.27 ± 0.02	15.71 ± 0.29	68.3 ± 3.8	114 ± 2
M8		40 ± 3	0.24	0.20 ± 0.03	12.15 ± 0.14	65.2 ± 6.6	106 ± 2
M9		60 ± 3	0.11	0.15 ± 0.02	18.27 ± 0.23	98.1 ± 5.9	11 5± 2
